# Treatment of Diseases of Infancy and Childhood

**Published:** 1883-11

**Authors:** 


**Treatment of Diseases of Infancy and Childhood,** with over four hundred formulæ and prescriptions, as exemplified in the services of Drs. Jacobs, Lewis, Smith, Alonzo Clark, Draper, Austin Flint, W. A. Hammond, W. L. Loomis, Guillard, Thomas, Leaming, Delafield, Agnew, Buckley, Lefferts and many others, and in the hospitals of New York City. By CHARLES H. GOODWIN, M. D. New York: C. H. Goodwin, M. D., 245 West Fifty-third Street. 1883. Price $2.50.

Something over a year ago, Dr. Goodwin published a volume on the treatment of diseases of the heart and lungs, and those of our readers who purchased it got the full value of their money, we are confident. The present volume is equally praiseworthy; it is arranged on the same plan, the practical therapeutical views of the several leading medical authorities of New York City being given; and that which, in their experience, has proved most valuable, the author has intelligently presented to his readers. We have no doubt the work will prove a useful one to the profession.

